# Evaluation of Neuraminidase Inhibitory Activity of Compounds and Extracts from Traditional Medicines by HPLC-FLD

**DOI:** 10.1155/2021/6694771

**Published:** 2021-08-24

**Authors:** Guangying Yu, Dan Fang

**Affiliations:** ^1^Department of Traditional Chinese Medicine, Tianjin Hospital, Tianjin 300211, China; ^2^Heping Hospital of No. 983 Hospital of Joint Service Support Force, Tianjin 300020, China

## Abstract

A simple and effective method was established and validated to determine 4-methylumbelliferone (4-MU) for screening the natural neuraminidase inhibitors (NAIs) from traditional medicines (TMs) by high performance liquid chromatography combined with fluorescence detection (HPLC-FLD). 4-MU and TMs compounds were separated on a Hedera TM ODS column (5 *μ*m, 4.6 × 250 mm) using an isocratic elution of 55% methanol at 35°C. The flow rate was 1 mL min^−1^. The excitation and emission wavelength were performed at 320 nm and 480 nm. Some extracts of TMs and compounds were selected as examples to demonstrate the feasibility of the new HPLC-FLD method. It was found that the results of most compounds except for the auto fluorescence substances determined by HPLC-FLD were in good agreement with NA enzyme-based inhibitory assays. Comparing to traditional NA enzyme-based inhibitory assays, the HPLC-FLD method could prevent interference from fluorescence pigments of compounds. It was considered a simple, effective, and economical technique for the screening the natural neuraminidase inhibitors from traditional medicines.

## 1. Introduction

Influenza has become a great public concern in recent years as a major health problem all over the world. The chemically synthesized neuraminidase inhibitors (NAIs) are widely used to prevent or treat influenza as antiviral drugs [1]. Zanamivir and oseltamivir are two common drugs introduced into clinical practice acting as NAIs, but clinical relevance of some mutations remains uncertain [2–4]. Fortunately, traditional medicines (TMs) and their preparations for clearing heat and detoxification have a very good therapeutic effect in the treatment of influenza in China [5, 6]. It was reported that some small molecules were identified as the potential natural neuraminidase inhibitors in *Radix Scutellariae* and Reduning injection (*Flos Lonicerae*, *Herba Artemisiae annuae*, and *Gardenia jasminoides* extract) [7, 8]. Traditional medicine resource is an important source of potential NAIs for antiviral drug screening. Because the chemical composition of TMs is complex, it is extremely necessary to establish a rapid screening method for the new, effective, and natural NAIs from TMs.

NA enzyme-based inhibitory assays are most widely used to evaluate the potential capabilities of compounds or extracts of TMs by fluorescence (FL) spectrophotometry. Recently, one-step high-performance liquid chromatography-fraction collector and time-of-flight mass spectrometry (HPLC-FC/Q-TOF-MS) or ultra performance liquid chromatography coupled with diode-array detectors and autofraction collector/time-of-flight mass spectrometry (UPLC-DAD-FC/QTOF-MS) coupled with bioactive assays were used to screen and identify natural NAIs from TM extracts [7, 8]. The affinity-based ultrafiltration UPLC-QTOF-MS analysis has been developed to identify potent bacterial NAIs from the plant extracts [9]. Among these reported methods, the compounds with autofluorescence such as flavonoids and coumarins in the screened samples may nonlinearly interfere with fluorescence measurements of 4-methylumbelliferone (4-MU) causing false positives or highly evaluated results [10]. To solve these problems, the neuraminidase-immobilized capillary microreactor has been applied to screen natural NAIs from traditional Chinese medicines. Nevertheless, this method has low sensitivity and reproducibility. These aforementioned events raise the need to explore a new method for screening natural NAIs from TMs.

The high-performance liquid chromatography combined with the fluorescence detection (HPLC-FLD) method is one of the commonly used separation methods of TMs and has been used in various operations. There are some reports about HPLC-FLD, including determination of the estrogenic hormones, amino acid, and aflatoxins [11–13]. It is sensitive and specific to avoid the false-positive results in NA enzyme-based inhibitory assays where some compounds are with autofluorescence. Hence, a new method HPLC-FLD was established to enhance advantages and avoid disadvantages.

The aim of the work was to establish a simple and effective method to determine 4-MU for screening the NAIs from TMs by HPLC-FLD. The HPLC-FLD method was suitable to screen the compounds from complex TMs which have potential capacity to inhibit NA. The interference from the screened compounds was eliminated by separating the components in samples, substrate, and product using HPLC-FLD. The positive drug peramivir was used to validate the assay as a reference inhibitor. Some types of compounds with or without autofluorescence such as coumarins, ridoids, flavonoids, and phenolic acids and four herbal medicines were selected to verify the feasibility of screening natural NAIs from TMs by the HPLC-FLD method. The HPLC-FLD method was highly efficient, more stable, economical, and feasible to screen NAIs compared with the result of the NA enzyme-based inhibitory assays. In addition, the new method established by HPLC-FLD can effectively avoid the false-positive result caused by some compounds with autofluorescence and improve the accuracy of the screening experiments.

## 2. Materials and Methods

### 2.1. Chemicals and Reagents

**Figure 1:**
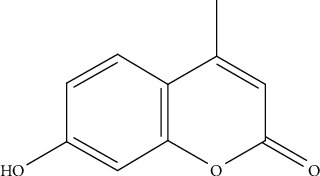
Chemical structure of 4-methylumbelliferone (4-MU).

The enzyme NA, the substrate 2′-(4-methylumbelliferyl)-*α*-*D*-N-acetylneuraminic acid sodium salthydrate (4-MuNeu5Ac), the product 4-MU (Figure 1), and positive drug peramivir were obtained from Sigma-Aldrich (Steinheim, Germany). Methanol (Dikma Technologies Inc, USA) were of HPLC grade. Deionized water was purified by using a Milli-Q academic water purification system (Millipore, Milford, MA, USA). The analytical reagents, sodium dihydrogen phosphate, and sodium hydrogen phosphate were purchased from Tianjin Kermel Science Company. The reference compounds of five ridoids (genipin, geniposide, shanzhiside methyl ester, geniposidic acid, and 8-O-acetyl shanzhiside methyl ester), five phenolic acids (chlorogenic acid, neochlorogenic acid, cryptochlorogenin acid, isochlorogenic acid A, and isochlorogenic acid C), two flavonoids (baicalin and baicalein), and nine coumarins (coumarin, notopterol, xanthotoxin, isoimpemtorin, psoralen, angelicin, xanthotol, bergapten, and osthole) were all purchased from Chengdu Dest Biotechnology Co., Ltd. (Chengdu, China).

### 2.2. Instrumentation

**Figure 2:**
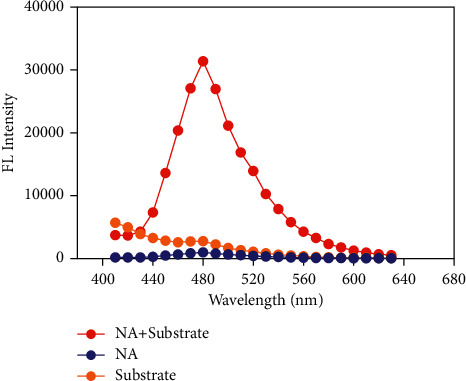
Fluorescence spectra under emission wavelengths at 410–630 nm and excitation at 320 nm.

The HPLC-FLD analysis was performed on an Agilent 1100 HPLC system with a fluorescence detector (FLD). The sample analysis was carried out on a Hedera TM ODS column (4.6 × 250 mm, 5 *μ*m) at 35°C with a flow rate of 1.0 mL min^−1^. The elution program was isocratic 16 min with 45% water and 55% methanol, and the injection volume was 10 *μ*L. According to the instructions of the Neuraminidase Inhibitors Screen Kit (Beyotime Biotechnology, P0309), the excitation and emission wavelengths of FLD were set at 320 nm and 480 nm, respectively (Figure 2).

### 2.3. Preparation of Solutions

#### 2.3.1. Preparation of Enzyme, Substrate, and Product Solutions

The buffer solution was prepared by adding a suitable amount of sodium dihydrogen phosphate to a moderate amount of sodium hydrogen phosphate until pH value reached 6.0. Stock solutions of NA and 4-MuNeu5Ac were dissolved in a phosphate buffer solution at the concentration of 25 U mL^−1^ and 10 *μ*g mL^−1^, respectively. Product 4-MU was prepared with methanol. All solutions were stored at −20°C.

#### 2.3.2. Preparation of Reference Compounds

The reference compounds of genipin, geniposide, shanzhiside methyl ester, geniposidic acid, 8-O-acetylshanzhiside methyl ester, chlorogenic acid, neochlorogenic acid, cryptochlorogenin acid, isochlorogenic acid A, isochlorogenic acid C, baicalin, baicalein, coumarin, notopterol, xanthotoxin, isoimpemtorin, imperatorin, psoralen, angelicin, xanthotol, bergaptenosthole, and peramivir were each prepared by dissolving with 50% methanol giving a stock concentration of 1.0 mg mL^−1^ and diluted into 8 different concentrations for bioassays and then stored at 4°C.

#### 2.3.3. Preparation of the Samples

*Scutellari abaicalensis, Notopterygium incisun, Gardenia jasminoides,* and *Lonicer japonica* were obtained from different markets and authenticated by Guangying Yu (Tianjin hospital). All herbal medicines were grounded into a powder in order to improve the extraction efficiency. *N. incisun* and *G. jasminoides* powder (10.00 g) were added into a 250 mL volumetric flask ultrasonically for 30 min and 20 min, respectively. *S. abaicalensis* powder (5.00 g) was dissolved in 500 mL with 70% methanol, and *L. japonica* (5.00 g) was prepared with 50% methanol. The two solutions were ultrasonic for 40 min and 30 min, respectively. Four herbal medicine extracts were combined, condensed, and dried by rotary evaporation. The extraction efficiency of *S. abaicalensis, N. incisun, G. jasminoides, and L. japonica* was 29.4%, 23.03%, 23.74%, and 28.2%, respectively. Then, all samples were stored at 4°C.

### 2.4. Method Validation

The HPLC-FLD method validation included linearity, limits of detection (LOD), limits of quantification (LOQ), and precision following the USFDA guidelines [14]. The stability and recovery tests were performed according to a previous reference [15]. Results obtained from the HPLC-FLD method were compared with those detected by NA enzyme-based inhibitory assays to demonstrate the feasibility of the novel method.

#### 2.4.1. Linearity, LOD, and LOQ

The calibration curve of 4-MU was constructed by plotting the peak area (*y*) against the corresponding concentration (*x*, ng mL^−1^). The limits of detection (LOD) and limits of quantification (LOQ) values were calculated at a signal-to-noise (*S/N*) ratio of 3 and 10 by diluting the highest concentration of the mixed solution further to a certain concentration, respectively.

#### 2.4.2. Precision, Stability, and Recovery

The analysis of intra- and interday precision were assessed by six repetitive injections of the standard solution at three different (low, medium, and high) concentrations in the same day and on three consecutive days, respectively. The precision was expressed as relative standard deviations (RSDs). The reference solution at three different concentrations was determined by replicate injection at 2, 4, 6, 8, 12, and 24 h to evaluate the stability. The recovery was calculated by comparing the experimental group with the controlled group. The experimental group solutions were prepared by spiking 10 *μ*L 4-MU at three different (low, medium, and high) concentrations into the centrifugal tubes with 10 *μ*L of 25 U mL^−1^ NA and 280 *μ*L phosphate buffer, respectively. The controlled group solutions consisted of 10 *μ*L 4-MU at three different (low, medium, and high) concentrations and 290 *μ*L phosphate buffer, respectively. All the solutions were in six replicates. Then, the mixed solutions were analyzed according to the conditions listed above. The recoveries at different concentrations of 4-MU were determined by the formula recovery (%) = (experimental group amount–controlled group amount) × 100%.

### 2.5. Bioassay

#### 2.5.1. Determination of Half Maximal Inhibitory Concentration (IC_50_)

The linear regression equation, expressed as *y* = *ax* + *b*, was used to determine the amount of product (4-MU) after the reaction of NA and 4-MuNeu5Ac. The experiments are conducted in 96-well plates. 70 *μ*L phosphate buffer, different amounts (2, 4, 6, 8, and 10 *μ*L) of NA at the concentration of 0.25 U mL^−1^, different amounts (10, 8, 6, 4, and 2) of phosphate buffer, and 10 *μ*L 50% methanol (because all the screened compounds were diluted with 50% methanol) were added to 96-well plates in order to obtain the linear regression equation of NA and product. To screen the compounds which can inhibit NA, 70 *μ*L phosphate buffer, 10 *μ*L NA at the concentration of 0.25 U mL^−1^, and 10 *μ*L of screening compound solutions at eight aliquots of serial suitable dilutions were added to 96-well plates in order. The samples were then mixed for 1 minute and incubated for 2 minutes. The last step was adding 10 *μ*L 4-MuNeu5Ac to each well at the concentration of 25 *μ*g mL^−1^ and shaking for 1 minute. After incubation for 30 minutes, the final fluorescence values were read by using a Flex Station 3 Microplate Reader [6]. All samples were in triplicate. The calculation of IC_50_ values was performed on the program GraphPad Prism 5.01. After fluorescence detection, 200 *μ*L methanols were added into each well and the 96-well plates were read by using a microplate reader again after 0, 5, 20, and 30 minutes to prove that the reaction was indeed terminated. Then, all solutions were pipetted into the centrifugal tubes and centrifuged for 10 min at 16650 g. The supernatant was injected in the HPLC. It is worth noting that calibration curves were carried out in each plate and each well must be repeated three times.

#### 2.5.2. *Km* Determinations

Neuraminidase (10 *μ*L), buffer solution (70 *μ*L), and ultrapure water (10 *μ*L) were added into 96-well fluorescent enzyme plates according to the abovementioned methods. Then, a series of concentrations (0–284 *μ*m) of neuraminidase fluorescent substrate (10 *μ*L) were added, vibrated, and mixed again for 1 min. After incubation at 37°C for 30 min, 200 *μ*L methanols were added into each well in the 96-well plates. Then, all solutions were pipetted into the centrifugal tubes and centrifuged for 10 min at 16650 g. The supernatant was injected in the HPLC. The *Km* value of neuraminidase was calculated by GraphPad prism software.

#### 2.5.3. *Ki* Determination

Neuraminidase (10 *μ*L) was mixed with a series of concentrations of peramivir (10 *μ*L, 0–457 *μ*m) and buffer solution (70 *μ*L), incubated at 37°C for 2 min, and then, added with a series of concentrations of the neuraminidase fluorescent substrate (10 *μ*L, 0–284 *μ*m). After incubation at 37°C for 30 min, the fluorescence was detected by HPLC-FLD. The *Ki* value of peramivir was calculated by GraphPad prism software.

#### 2.5.4. Statistical Analysis

All analysis of the bioassay experiments was measured in triplicates. The values were expressed in the form mean ± standard deviation (SD); IC_50_ values were calculated by the program GraphPad Prism 5.01.

## 3. Results and Discussion

### 3.1. Optimization of HPLC Separation Conditions

**Figure 3:**
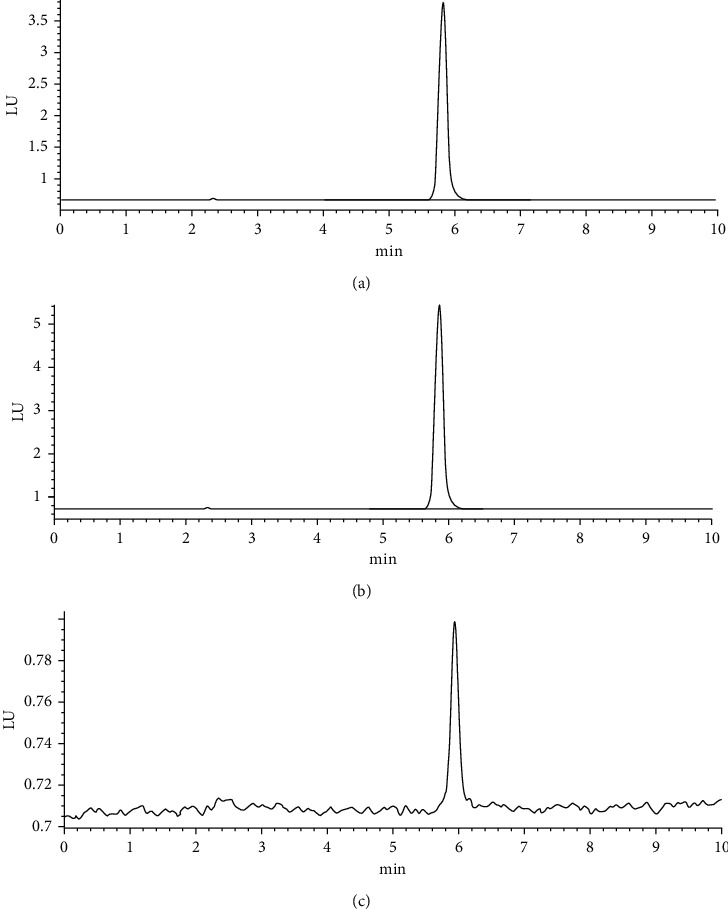
The typical chromatogram: (a) reference compounds of 4-methylumbelliferone, (b) product of 4-methylumbelliferone, and (c) product of 4-methylumbelliferone inhibited with baicalin or natural product extracts.

The substrate including 22 screened compounds and peramivir was separated from the product on a Hedera TM ODS column (4.6 × 250 mm, 5 *μ*m). The mobile phase composition, different flow rates, and column temperatures were investigated in order to achieve good resolution and symmetrical peak shape in a shorter analysis time. The mobile-phase compositions were prepared by different concentrations of methanol and acetonitrile. The optimum conditions were as follows: isocratic elution with 55% methanol for 16 min. In addition, column temperatures (25, 35, and 40°C) and flow rates (0.8, 1.0, and 1.2 mL min^−1^) were also investigated. Finally, column temperature and flow rate were optimal at 35°C and 1.0 mL min^−1^, respectively. The result depicted in Figure 3 suggested that the product was 4-methylumbelliferone. Moreover, the HPLC-FLD method could be applied to screen NAIs due to the decreased fluorescence value of the product after reaction.

### 3.2. Method Validation

#### 3.2.1. Linearity, LODs, and LOQs

The linear regression equation of 4-methylumbelliferone (product 4-MU) is as follows: *Y* = 0.6608 *x* + 0.1247. The linear range ranged from 0.16 ng mL^−1^ to 100 ng mL^−1^. A good linear relationship was obtained with *r*^2^ = 0.9997. The LOD and LOQ value of 4-methylumbelliferone were 0.05 ng mL^−1^ and 0.16 ng mL^−1^, respectively. These results demonstrated that HPLC-FLD was highly sensitive in the determination of the product 4-methylumbelliferone.

#### 3.2.2. Precision, Stability, and Recovery

The intraday and interday precision at different concentrations (low, medium, and high) are shown in Table 1. Their RSD values were less than 2.66% and 4.64%, respectively. The stability of 4-MU was analyzed within one day at 0, 2, 4, 6, 8, 12, and 24 h, the RSD values ranged from 2.95% to 4.88%, and the range of accuracy was 102% to 105%. The RSD values and recoveries were in the range of 1.38% to 2.58% and 100% to 102%, respectively (Table 2).

### 3.3. Bioassay

#### 3.3.1. Optimization of the Proportion of Enzyme and Substrate

**Figure 4:**
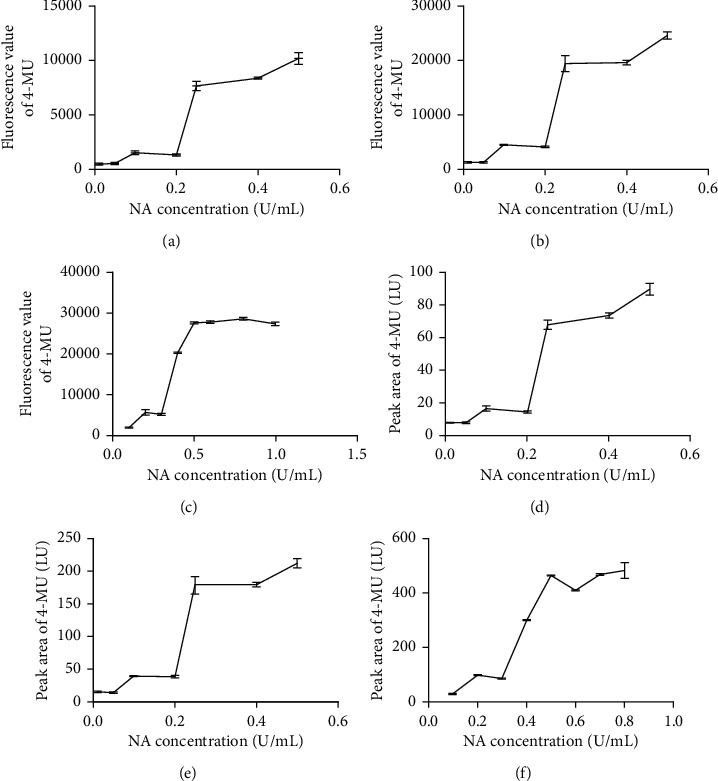
The effect of different NA concentrations and substrates on the fluorescence value and peak area of 4-MU by NA enzyme-based inhibitory assays and HPLC-FLD, respectively. (a) The effect of 10 *μ*g mL^−1^ substrate on the fluorescence value of 4-MU, (b) the effect of 25 *μ*g mL^−1^ substrate on the fluorescence value of 4-MU, (c) the effect of 50 *μ*g mL^−1^ substrate on the fluorescence value of 4-MU, (d) the effect of 10 *μ*g mL^−1^ substrate on the peak area of 4-MU, (e) the effect of 25 *μ*g mL^−1^ substrate on the peak area of 4-MU, and (f) the effect of 50 *μ*g mL^−1^ substrate on the peak area of 4-MU.

Taking different proportions of NA and substrate into account, various concentrations of NA were reacted with a certain concentration of substrate each time. Three concentrations (10 *μ*g mL^−1^, 25 *μ*g mL^−1^, and 50 *μ*g mL^−1^) of the substrate were investigated to evaluate the effect of different proportions on the fluorescence value and peak area of 4-MU, respectively. Figures 4(a) and 4(d) implied that the fluorescence values and peak areas of 4-MU increased linearly lower than 0.25 U mL^−1^ of NA. However, the trend curve was slowed down after concentration. Thus, the 0.25 U mL^−1^ of NA was chosen the optimum condition reacted with 10 *μ*g mL^−1^ of 4-MU. When the concentration of the substrate was 25 *μ*g mL^−1^, the result of optimization was as displayed in Figures 4(b) and 4(e)). The variation tendency tended to flatten out at the concentration of 0.25 U mL^−1^ of NA. 50 *μ*g mL^−1^ of the substrate was also assayed, and the result is listed in Figures 4(c) and 4(f)). The fluorescence values and peak areas of 4-MU increased significantly with the increase in the concentration lower than 0.5 U mL^−1^ of NA.

When the concentrations of the substrate were 10 *μ*g mL^−1^, 25 *μ*g mL^−1^, and 50 *μ*g mL^−1^, the concentrations of NA were optimum at 0.25 U mL^−1^, 0.25 U mL^−1^, and 0.5 U mL^−1^, respectively. It was found that there is no significant difference between the results determined by NA enzyme-based inhibitory assays and HPLC-FLD. In the experimental process, it is a fact that higher concentrations of substrate and NA result in lower inhibitory potential of the samples at the same concentration. In the contrary, the concentrations of the substrate and NA were lower; the ability of inhibition would be highly evaluated. Finally, the condition of 10 *μ*g mL^−1^ of the substrate and 0.25 U mL^−1^ NA was achieved to screen the inhibitors from TMs.

#### 3.3.2. Inhibitor Screening

In order to validate the accuracy of the enzyme activity, the positive drug peramivir was selected to test the half-maximal inhibitory concentration (IC_50_) by both NA enzyme-based inhibitory assays and the HPLC-FLD method. The results showed that the *Km* value of neuraminidase was 93.23 ± 9.03 *μ*m and the *Ki* value of peramivir was 47.01 ± 2.54 *μ*m. IC50 of peramivir was 290 ± 30 *μ*m by NA enzyme-based inhibitory assays and 270 ± 40 *μ*m by the HPLC-FLD method. It was demonstrated that the method to determine 4-methylumbelliferone (4-MU) by HPLC-FLD could accurately be used to determine the inhibitory activity of compounds on neuraminidase. Twenty-one natural compounds were determined by new methods. It was found that the NA inhibitory activity of seven compounds (genipin, geniposidic acid, 8-O-acetyl shanzhiside methyl ester, neochlorogenic acid, coumarin, notopterol, and xanthotoxin) was lower than 50% at test concentration (500 *μ*g mL^−1^). It was shown that these seven compounds have weak inhibitory activity on neuraminidase. The results in Table 3 displayed the IC_50_ values of other 14 compounds (geniposide, Shanzhiside methyl ester, chlorogenic acid, cryptochlorogenin acid, isochlorogenic acid A, isochlorogenic acid C, baicalin, baicalein, isoimpemtorin, psoralen, angelicin, xanthotol, bergapten, and osthole). The results indicated that fourteen natural compounds showed potential inhibition for NA. The inhibition potentials were ranked in the following order: shanzhiside methyl ester > baicalein > peramivir > baicalin > isoimpemtorin > psoralen > isochlorogenic acid A > xanthotol > isochlorogenic acid C > geniposide > chlorogenic acid > angelicin > osthole > cryptochlorogenin acid > bergapten. The ranking order of the inhibition potency of baicalein > baicalin was consistent with that shown in a previous paper [16]. Four TMs of *S. baicalensis, N. incisun, G. jasminoides,* and *L. japonica* were further used to screen inhibitory activity due to four TM extracts having the 21 components screened above, respectively. All extracts were diluted into 10 different concentrations to calculate the IC_50_ values. The result displayed in Table 4 suggested that the four TMs extracts have good inhibitory activity to NA. The experimental results agreed with those in [17] except the *G. jasminoides* because there is no study published about it. *S. baicalensis, N. incisun,* and *L. japonica* extracts exhibited a significant inhibitory activity to NA at 40 *μ*g mL^−1^. However, the IC_50_ values of the three extracts were 218 *μ*g mL^−1^, 4.44 *μ*g mL^−1^, and 1392 *μ*g mL^−1^, respectively. The different IC_50_ values of the two studies might be caused by assaying with different systems. In summary, the facts that the four extracts of TCMs were highly effective to inhibit NA activity have been further confirmed. It was concluded that HPLC-FLD method was highly efficient, more stable, economical, and feasible to simultaneously screen NAIs from compounds and extract from TMs.

## 4. Conclusions

A simple and effective HPLC-FLD method was established and validated to determining 4-methylumbelliferon for screening the natural neuraminidase inhibitors (NAIs) from traditional medicines. Compared with the results of NA enzyme-based inhibitory assays, the method was sensitivity, effective, and economical accounting for the extremely low LOD and LOQ values, high stability, and good accuracy. Additionally, the novel method can greatly minimize the false-positive results caused by the autofluorescence substances in NA enzyme-based inhibitory assays. It was concluded that the new method for determining 4-methylumbelliferone by HPLC-FLD is a promising screening tool for NAI traditional medicines.

## Figures and Tables

**Table 1:** Intraday and interday accuracy and precision and stability of 4-methylumbelliferone (*n* = 6).

Compound	Concentration (ng/mL)	Interday		Intraday		Stability	
		RSD (%)	Accuracy (%)	RSD (%)	Accuracy (%)	RSD (%)	Accuracy (%)
4-Methylumbelliferone	0.50	2.26	99.8	3.25	102	4.88	102
	5.00	1.92	99.6	4.56	102	3.81	105
	50.00	1.92	102	4.64	98.3	2.95	105

**Table 2:** The recovery of 4-methylumbelliferone (*n* = 3).

Compound	Concentration (ng/mL)	Recovery	
		RSD (%)	Accuracy (%)
4-Methylumbelliferone	0.50	2.01	102
	5.00	1.38	100
	50.00	2.58	100

**Table 3:** The IC_50_ values of 15 compounds assayed with NA enzyme-based inhibitory assays and the HPLC-FLD method (mM).

No.	Compounds	NA enzyme-based inhibitory assays	HPLC-FLD
1	Geniposide	1.01 ± 0.09	0.97 ± 0.09
2	Shanzhiside methyl ester	0.01 ± 0.00	0.01 ± 0.00
3	Chlorogenic acid	1.39 ± 0.13	2.56 ± 0.06
4	Cryptochlorogenin acid	1.59 ± 0.01	2.20 ± 0.01
5	Isochlorogenic acid A	0.71 ± 0.01	1.11 ± 0.07
6	Isochlorogenic acid C	0.80 ± 0.03	1.17 ± 0.05
7	Baicalin	0.32 ± 0.04	0.50 ± 0.04
8	Baicalein	0.06 ± 0.02	0.11 ± 0.01
9	Isoimpemtorin	0.43 ± 0.03	0.66 ± 0.16
10	Psoralen	0.70 ± 0.06	1.00 ± 0.63
11	Angelicin	1.46 ± 0.31	2.55 ± 0.38
12	Xanthotol	0.76 ± 0.07	1.05 ± 0.07
13	Bergapten	2.05 ± 0.20	2.80 ± 0.16
14	Osthole	1.50 ± 0.18	2.14 ± 0.15
15	Peramivir	0.29 ± 0.03	0.27 ± 0.04

**Table 4:** The IC_50_ values of 4 extracts assayed with NA enzyme-based inhibitory assays and the HPLC-FLD method.

Extracts	IC_50_ (*μ*g/mL)	
	NA enzyme-based inhibitory assays	HPLC-FLD
*S. baicalensis*	187 ± 10	218 ± 19
*N. incisun*	3.53 ± 0.58	4.44 ± 0.91
*G. jasminoides*	2.37 ± 0.60	3.22 ± 0.59
*L. japonica*	979 ± 77	1392 ± 100

*Note.* The extraction efficiency of *S. abaicalensis, N. incisun, G. jasminoides,* and *L. japonica* was 29.4%, 23.03%, 23.74%, and 28.2%, respectively.

## Data Availability

The data used to support the findings of this study are included in this article.
